# A Christmas Appeal for the Hospitals

**Published:** 1921-12

**Authors:** 


### A CHRISTMAS APPEAL FOR THE LONDON HOSPITALS.

FOLLOWING the example of "The Hospital," its predecessor and parent, this journal makes in its December issue a special appeal on behalf of the hospitals.

Readers of THE HOSPITAL AND HEALTH REVIEW hardly need reminding of the magnificent work of the voluntary hospitals, not only on the charitable side, which is most obvious to the lay public, but in their services to research and as schools of training in medicine and nursing. As they, better than most people, are aware, the position of the hospitals is a serious, almost a desperate, one. It is not that public support has declined since the beginning of the war, but that the 1921 guinea goes no further than the 1913 half-sovereign, and that the difference is too great to be made up by any ordinary effort.

Until within the last few years it was possible for each hospital, with the aid of its special friends, to hold its own independently of the rest of the hospital world. Under the conditions brought about by the war it is no longer a question of the welfare of individual institutions, but of the survival of the voluntary principle itself. We have not yet sufficiently apprehended, perhaps, the greatest of the lessons which we should have learnt from the war—that only by the united efforts of the whole community can anything affecting the community be accomplished.

The Local Voluntary Hospitals Committee of King Edward's Hospital Fund estimates this year's aggregate deficit at £360,000 for the maintenance account of the London hospitals alone. From the Government grant of half-a-million for the whole of Great Britain, £180,000 is provisionally appropriated for London, of which emergency grants amounting to £77,900 are to be made at once to those hospitals which are in most urgent need. To others which are able to carry on for the present no emergency grants are yet allowed. The total amount available for distribution represents only 50 per cent. of the estimated deficit, and is conditional on the raising of a corresponding sum by the hospitals themselves. Therefore every pound now contributed to the funds of any hospital is twice blest, in that it not only swells the funds of the institution but enables it to earn another pound. King Edward's Hospital Fund itself is entitled to the support of those who have no favourite local or other hospital to claim their help. Donors to the Fund may be assured that their contributions will be well and wisely expended.

It must not be forgotten that this sum of £180,000 merely represents the minimum which the London hospitals must have in order to carry on, on the present basis. Nothing is allowed for new buildings and extensions, however desirable.

If every householder would become a hospital subscriber, if only to the extent of an annual guinea; if every subscriber would add a trifle to his present gift; if every family would take a keener interest in the welfare of the collecting-box, which is, or should be, a cherished institution in the home, hospital finance would soon be in a sound condition. In this number we publish a few details of some of the needs of hospitals in the Metropolis. In our January issue we intend to give further information about other London hospitals.

***We shall be pleased to receive for publication in our next issue a short statement of the immediate and future needs of any Hospital that is not mentioned in the following list. Communications on this subject should be addressed to the Editor of "The Hospital and Health Review," and should arrive at this Office not later than December 12, 1921.—Editor, H. & H. R.***

### HOSPITALS WITH 150 BEDS AND UPWARDS.

**City of London Hospital for Diseases of the Chest.**
Expenditure nearly double that of 1914. The maintenance of the Sanatorium of twenty beds at Saunderton, Bucks, requires additional income this year of about £1,000. Sanitary and other improvements approved by King Edward's Hospital Fund are urgently necessary. Help to the extent of a minimum of £5,000 is earnestly sought.

**Great Northern Central Hospital.**
Two hundred urgent cases are awaiting admission. £18,000 required before December 31. Must close some wards unless additional funds are immediately provided.

**Guy's Hospital.**
A special financial difficulty is to repay the large capital sums which have been employed in building extensions and improvements which could no longer be delayed. Annual expenditure £150,000; assured income only £60,000.

**Hospital for Sick Children.**
An annual deficit between assured income and total expenditure of some £16,000 and a debt of £35,000. Unless substantial help is forthcoming must reduce work or close the hospital before the spring of 1922.

**King's College Hospital.**
At present has a debt of £100,000 and tradesmen's accounts to the extent of about £20,000 remain unpaid. In the early part of the year the Committee of Management was compelled to close 160 beds in order to avert a worse disaster. The total cost of maintaining the Institution, with its 400 beds in full use and the various special departments, is about £131,000 a year. There is an assured income of about £51,000 per annum, including patients' contributions. The Institution earnestly appeals for an additional £80,000 a year that it may be maintained in a state of efficiency.

**London Homœopathic Hospital.**
Must secure another £5,000 per annum in order to make provision for barest necessities, or must curtail services.

**London Hospital.**
Has a debt of £40,000 to reduce; 200 beds remain closed; reductions have had to be made in the number of out-patients treated.

**National Hospital for the Paralysed and Epileptic.**
Expenditure has risen to about £40,000 a year, of which £18,000 is paid by, or on behalf of, the patients. Income, however, will not meet expenditure this year by at least £5,000.

**A Christmas Appeal for London Hospitals—(*cont.*).**

**St. George's Hospital.**

Ordinary expenditure in 1920 exceeded ordinary income by over £35,000, and there is a similar deficit this year. Donations specially earmarked towards the cost of the new Physio-Therapy Department, and towards the replacement of furniture in the nursing staff's quarters, would be welcome.

**St. Thomas's Hospital.**

In 1914 the income of the Hospital was sufficient to meet expenditure, but in 1921 the cost of maintenance has increased so enormously that three of the wards have had to be closed. Almost every patient willingly contributes who can afford to do so, and over 120 firms are now making weekly collections amongst their workmen for the support of the Hospital; but even these efforts to increase the income have proved insufficient. The Hospital is, unfortunately, burdened with a heavy debt which accumulated during the period of the war, when 11,376 soldiers, almost entirely from service abroad, were treated, in addition to the ordinary civilian patients.

**Westminster Hospital.**

£8,500 urgently needed for a properly equipped Physio-health from the classes using the hospital. Annual deficit £15,000. May have to curtail its work unless further help is forthcoming before the end of the year.

### HOSPITALS WITH OVER 100 AND UNDER 150 BEDS.

**Prince of Wales's General Hospital, Tottenham.**

Debt of £14,000 on account of maintenance, liquidation of which would give many advantages and economies in working. Serves a very poor and populous neighbourhood in North-East London and is seriously hampered by limited accommodation.

**Victoria Hospital for Children.**

£8,500 urgently needed for a properly equipped Physio-Therapy Department and further accommodation for the Out-patient Department. Has also a deficit of £8,000 this year. For the renewal of the central heating plant and other improvements £15,000 is required.

### HOSPITALS WITH 50 TO 100 BEDS.

**Chelsea Hospital for Women.**

Work much restricted, as half the wards have to be used to house the nursing staff until the Nurses' Home is built. For this purpose a further £12,500 is required. There is a debt on the General Fund of £4,500.

**City of London Maternity Hospital.**

Oldest Maternity Hospital in the kingdom (established 1750). Ante-natal Centre and Infant-welfare Centre much hampered in their work by inadequate accommodation, and improvement is the great need of the moment. Present indebtedness to bankers is £5,850.

**Queen Charlotte's Lying-in Hospital.**

Receives into its wards nearly 2,000 patients every year, and attends over 2,000 patients in their own homes. Also has large ante-natal and child-welfare departments. In debt to the extent of about £10,000, and cannot proceed with a much-needed extension, including a new out-patient department.

**St. Andrew's Hospital, Dollis Hill Lane.**

Has a heavy deficit on its maintenance account and a debt on the building. One of the few hospitals catering for gentlepeople and the middle classes.

**West London Hospital for Nervous Diseases.**

Recently compelled to reduce number of beds from 102 to 62.

### HOSPITAL WITH UNDER 50 BEDS.

**Paddington Green Children's Hospital.**

In urgent need of funds to pay off loan of £7,300 from its bankers.

### GENERAL CHARITIES.

**Dr. Barnardo's Homes, Stepney Causeway.**

One of the worthiest and best-loved charities in this country. The institutions now comprise 156 separate branches and homes. No destitute child is ever refused admission. In 1920 1,413 children were admitted; 14,497 dealt with; 7,280 actually under care of the Homes. Expenses of maintenance have greatly increased. It is not generally remembered that the expenditure of Her Majesty's Hospital, Stepney Causeway, with sixty-six beds, is defrayed by this charity.

**Royal Dental Hospital of London.**

Mortgage on building £20,957, the interest on which is in itself a heavy burden. Present bank overdraft, £1,514; outstanding accounts, £3,000.

**London Spectacle Mission.**

Provided spectacles last year for nearly 2,000 poor and aged people unable to procure them for themselves. The cost of spectacles has risen fourfold. Appeals for special gifts and donations during the present hard times, and for gifts of disused glasses suitable for normal failing sight.

---

### THE POUND-FOR-POUND PRINCIPLE.

As we go to press the Voluntary Hospitals Commission of the Ministry of Health sends us for publication the following correspondence regarding the condition that fresh money shall be raised on a pound-for-pound basis from voluntary sources:—

On behalf of the Commission, Lord Onslow wrote to the Chancellor of the Exchequer, under date November 16:—

> The Voluntary Hospitals Commission have asked me to forward for your consideration the accompanying resolution received from King Edward's Hospital Fund, who act as the Local Hospital Committee for London. In view of the fact that the stipulation to which the resolution refers is specifically set out in the terms of their appointment, the Commission feel themselves precluded from offering any observations in regard to it.

The resolution referred to, which was taken at a meeting of the President and General Council of King Edward's Hospital Fund, held at St. James's Palace on November 9, was as follows:—

> That the President and General Council of King Edward's Hospital Fund for London have considered the condition attached by the Treasury to the temporary assistance voted by Parliament to voluntary hospitals requiring that fresh money shall be raised pound for pound with the Government grants, and are of opinion that the application of this condition, which was not recommended in the report of Lord Cave's Committee, nor separately considered (much less expressly enjoined) by Parliament, will lead to great difficulties and probably to great injustices as between different hospitals in the distribution of the grants; and they trust, therefore, that the condition will not be pressed.

Sir Robert Horne replied to Lord Onslow, under date November 18:—

"The stipulation that the Government grant of £500,000 should be administered by your Commission on a pound-for-pound basis, to which the resolution in question takes exception, is, of course, absolutely fundamental. Furthermore, and particularly having regard to the present financial position, I wish to make it clear beyond any possibility of doubt that the £500,000 grant is the limit of Government assistance to the voluntary hospitals. It is of great importance that the position should be clearly understood both by the King Edward's Hospital Fund and local hospital committees generally, otherwise any appeal for fresh money from the public may meet with disappointing results."

[We publish on p. 89 an article on this decision, and we intend to comment on the matter again in our next issue.—Ed., *H. & H. R.*]

